# Case report—Depression with psychotic features as an atypical presentation of neurosyphilis

**DOI:** 10.1002/ccr3.8836

**Published:** 2024-04-30

**Authors:** Aykut Aytulun, Laura Sophie Grannemann, Colin R. MacKenzie, Alexandros Dimitrakopoulos, Francesca Pessanha‐Schlegel, Daniel Kamp, Leonhard Schilbach

**Affiliations:** ^1^ Department of General Psychiatry 2, Medical Faculty, LVR‐Klinikum Düsseldorf Heinrich‐Heine University Düsseldorf Düsseldorf Germany; ^2^ Department of Microbiology Heinrich‐Heine‐University Düsseldorf Düsseldorf Germany; ^3^ Medical Faculty Ludwig‐Maximilians‐Universität Munich Germany

**Keywords:** cerebrospinal fluid, depression with psychotic features, neuropsychiatry, neurosyphilis

## Abstract

Recurrent depression with psychotic features is an atypical presentation of neurosyphilis. This case emphasizes the polymorphic clinical presentation of neurosyphilis and how it mimics affective disorders with psychotic symptoms.

## Graphical abstract

Timeline of treatment, symptoms, and medication over eight years since first admission.

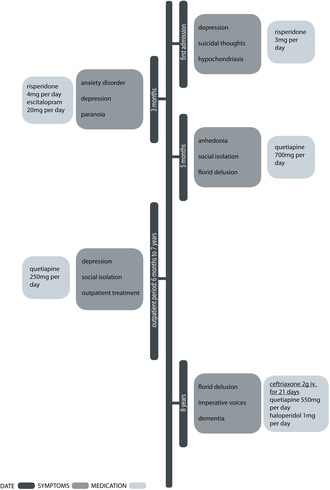

## 1 INTRODUCTION

Neurosyphilis is a rare complication caused by the spirochete *Treponema pallidum* ssp. *pallidum*. Possible neurological symptoms include ataxia, headache, cranial nerve palsy, impaired vision, and hearing loss. Furthermore, neurosyphilis may cause psychiatric symptoms such as delusion, disorientation and hallucination, depression, anxiety, personality changes, and cognitive decline.^1^ Due to the wide range of symptoms of neurosyphilis, Sir William Osler coined the term “the great imitator.”^2^

Before the advent of antibiotics, a large proportion of psychiatric in‐patients (up to 20%) suffered from neurosyphilis.^3^ With the introduction of penicillin, the prevalence of neurosyphilis decreased rapidly in the 1940s.^1^ Recently, there has been a growing number of neurosyphilis cases worldwide^4^ including Germany.^5^

The diagnosis of neurosyphilis is challenging and requires the examination of the cerebrospinal fluid (CSF). A diagnosis of syphilis is established by a positive serology of Treponema pallidum in an Hemagglutination Assay (TPHA), a pleocytosis in the CSF as well as a high protein fraction indicating a blood brain barrier dysfunction and IgM or IgG synthesis in the CSF.^6^ There are no sensitive serological tests that definitively diagnose neurosyphilis. The presence of a positive anti‐cardiolipin antibody test establishes the diagnosis of neurosyphilis; however, this test has a sensitivity of only 50%.^7^

Here, we report on a case of neurosyphilis that remained undiagnosed for nearly a decade with a clinical presentation that included solely affective and psychotic symptoms.

## 2 CASE HISTORY/EXAMINATION

A 63‐year‐old man was admitted to our inpatient ward due to his depression. In addition, the patient reported auditory hallucinations consisting of imperative voices instructing him to remain in bed. Furthermore, he suffered from delusions of persecution and poisoning. The patient reported that he was being observed by unknown people that wanted to hurt him, he also reported, that his wife poisoned his food. The family members reported that he lived in social isolation.

On physical examination the skin was normal, the patient was afebrile and there were no clinical signs of infection. The examination of heart, lungs, and abdomen did not reveal any pathological findings. On neurological examination the cranial nerves and especially pupillary reflexes were normal, there was no evidence of weakness, rigor, or bradykinesia. There were no sensory deficits. Deep tendon reflexes were brisk, plantar reflexes were negative, and his gait was normal.

## 3 DIFFERENTIAL DIAGNOSIS, INVESTIGATIONS, AND TREATMENT

The complete blood count, C‐reactive protein, liver function tests, electrolytes, creatinine, urea, sodium, potassium, calcium, urine drug screening, and thyroid function tests were within normal limits. Serological testing for infectious causes was not performed. Due to the presence of psychotic features a MRI of the head was performed, which ruled out structural pathologies. Assuming a depression with psychotic features, we initiated a treatment with risperidone 3 mg and escitalopram 20 mg daily under which the psychotic features remitted but re‐occurred after 3 months. The daily dose of risperidone was increased to 4 mg and after no significant improvement, risperidone was switched to quetiapine 700 mg daily. Quetiapine has dual antidepressant and antipsychotic effects. Escitalopram was discontinued to improve medical adherence with the quetiapine monotherapy. The psychotic features showed full remission, but the depression did only remit partially.

One year later the patient retired from his work. After discharge from the hospital, the patient visited the out‐patient psychiatrist infrequently. Quetiapine was reduced to 250 mg daily due to the remission of psychotic features.

Eight years after first admission, the family noticed a change of mental state characterized by disorientation, cognitive decline, and reappearance of psychotic symptoms over a period of 2 months, so that the patient was no longer able to live on his own.

**FIGURE 1:**
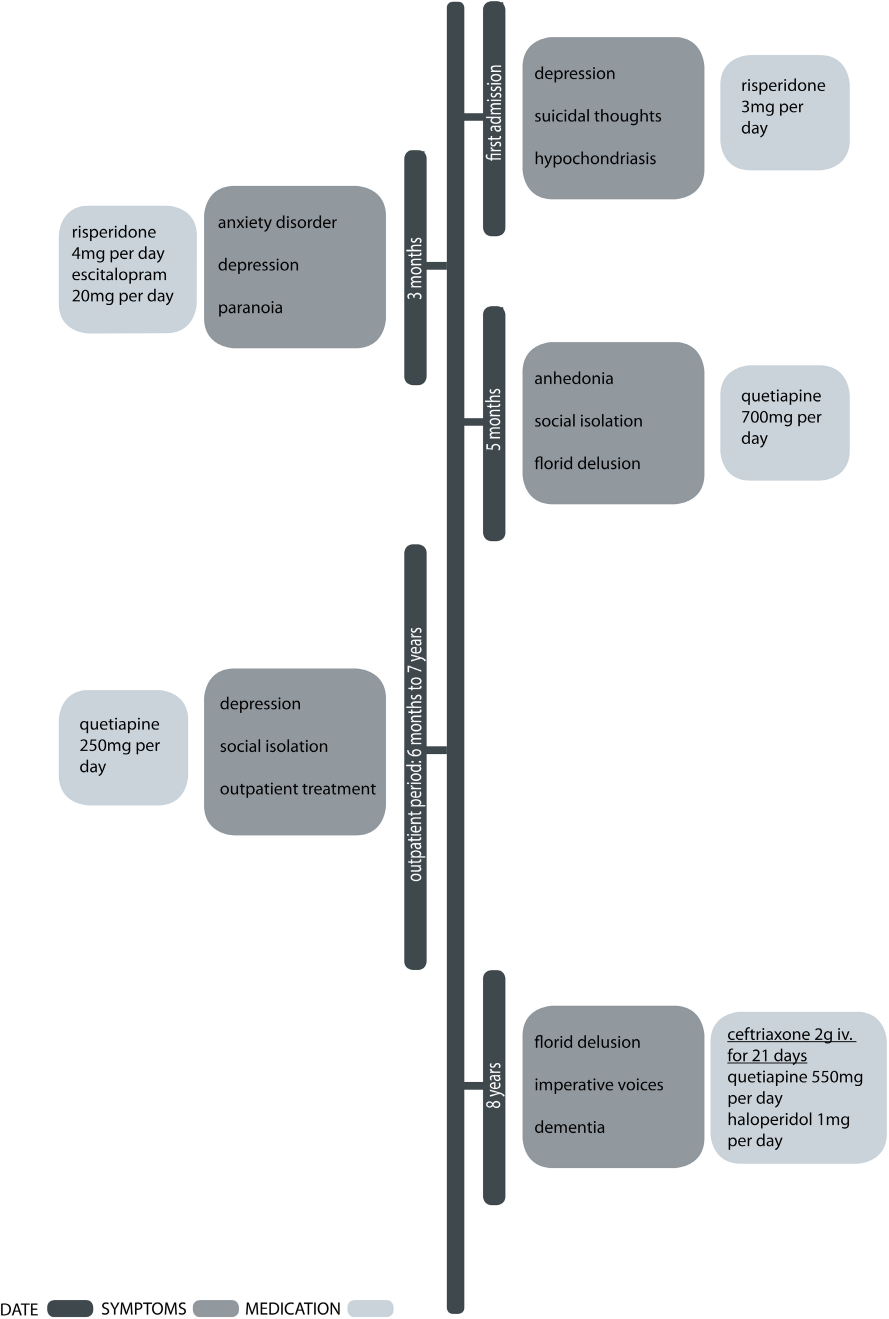
Timeline of treatments including symptoms and medication.

The patient was readmitted and displayed a depressive syndrome, including anhedonia, social isolation, and loss of appetite. The family reported psychotic symptoms, with the patient believing a camera was implanted in his right arm during recent surgery for a radius fracture. Similar to the previous admission, he continued to hear imperative voices that claimed he was “guilty.” The circadian rhythm was disturbed, and the patient behaved erratically such as rolling himself into a carpet or undressing in the courtyard of the hospital. The timeline of the treatment is summarized in Figure 1. The examination showed no neurological deficits, especially no evidence for tabes dorsalis, Argyl‐Robertson‐pupils, and the cranial nerves were not affected.

A review of his medical history revealed that the patient had an ulceration of his penis more than 15 years ago, which resolved spontaneously without treatment.

Considering this report, testing for syphilis revealed a positive serum TPHA with a titer of 1:81,920. Consequently, the spinal tap showed a pleocytosis of 8 cells/μL, elevated protein of 1252 mg/L consistent with a blood brain barrier disturbance, positive oligoclonal bands and IgM and IgG synthesis. The TPHA titer in the cerebrospinal fluid was 1:4096 with a positive serum to cerebrospinal fluid index of 3.5. The anti‐cardiolipin antibody titer was 1:64 in serum. Chemokine (C‐X‐C motif) ligand 13 (CXCL 13) was elevated with 199.6 pg/mL. Antineuronal antibodies were negative in serum and cerebrospinal fluid. The neurodegenerative markers (total tau protein, phosphorylated tau protein, β‐amyloid 1–42, and β‐Amyloid 42/40 ratio in cerebrospinal fluid) were not indicative of dementia. The serology was negative for the human immunodeficiency virus (HIV) and hepatitis C and showed a resolved hepatitis B infection (HBsAg negative, anti‐HBs negative, anti‐HBc positive, and hepatitis B virus DNA negative). The patient scored 22/30 points on the mini‐mental state examination, which is consistent with mild dementia. The Positive and Negative Syndrome Scale (PANSS) showed 26 points on the positive scale, 31 on the negative scale, and 58 points on the general psychopathology scale with a total of 115 points. The Hamilton Depression Scale revealed a severe depression (43 of 66 points). There was no evidence of luetic lesions in the patients' ears and eyes. A follow‐up MRI of the head showed only mild microangiopathy without signs of vasculitis or stroke, and no relevant changes compared with the MRI performed at first admission. A spinal MRI showed no features consistent with luetic gumma. The relatives reported that the patient had never been diagnosed for syphilis. Based on this information, we attributed previous psychiatric symptoms, their progression and the cognitive decline to an untreated neurosyphilis. The persistence of affective and psychotic symptoms despite pharmacological treatment is noteworthy.

The patient was treated intravenously with ceftriaxone for 21 days. During this treatment, the patient initially worsened with sexual disinhibition, disorganized behavior and psychomotor agitation.

We added haloperidol 1 mg to enhance dopamine 2 receptor blockade and increased quetiapine to 550 mg daily.

## 4 OUTCOME AND FOLLOW‐UP

Five days after completion of intravenous anti‐infective treatment with ceftriaxone the patient was discharged against medical advice in the care of family members (with the son being the legal guardian) in partial remission. He was still experiencing auditory hallucinations, but he was able to distinguish them as unreal. The cognitive deficits persisted, and he needed help with daily activities, consistent with a dementia caused by neurosyphilis. To assure the effectiveness of the antibiotic treatment, we recommended a lumbar puncture in 6 months. His out‐patient psychiatrist was informed about the current diagnosis and the necessary follow‐ups.

## 5 DISCUSSION

This case demonstrates the importance of a thorough diagnostic work‐up in all patients presenting with fluctuating and persisting psychiatric symptoms, especially with a late onset. An atypical clinical presentation or worsening are red flags for an organic etiology.

We diagnosed a patient with depression and psychotic features, which is important to distinguish from unipolar depression. Furthermore, the patient developed signs of disorientation and cognitive decline, while the MRI of the head showed no regional atrophy or cerebral microangiopathy suggestive of a vascular dementia.

A routine serum screening for syphilis should be performed as psychiatric symptoms as the sole clinical manifestation of neurosyphilis are quite common.^8^ This emphasizes that psychiatrists must bear this differential diagnosis in mind.^9^ Because of the patient's delusion of poisoning and suspicion toward the medical staff, we treated him with a single dose of ceftriaxone instead of five doses of penicillin daily, as a recent study has shown no difference between both regimens in the treatment of neurosyphilis in psychiatric patients.^10^ In this case, it is difficult to classify the syphilis stage as the history is not specific enough. As symptoms began approximately 9 years ago, we assumed a late‐stage neurosyphilis. It is also not uncommon that late‐stage neurosyphilis presents without parenchymatous lesions of the brain (gumma).^11^

We interpreted the initial worsening of the psychiatric symptoms during the treatment with ceftriaxone as a form of Jarisch‐Herxheimer reaction.^12^ A prophylactic treatment with cortisone is not considered routine, as the reaction resolved quickly requiring only supportive measures. The positive outcome is linked to an early diagnosis. Severely affected individuals are still capable of considerable benefit.^13^ In patients over 60 years, however, remission is less frequent.^14^ The elevation of CXCL13 is considered as a biomarker for the disease activity of neurosyphilis.^15^

Due to increasing burden of syphilis in Germany especially in large cities such as Cologne, Munich, and Berlin,^5^ we recommend a routine serum testing for syphilis in psychiatric patients.

## AUTHOR CONTRIBUTIONS

**Aykut Aytulun:** Conceptualization; formal analysis; investigation; project administration; validation; writing – original draft; writing – review and editing. **Laura Sophie Grannemann:** Conceptualization; data curation; investigation; project administration; validation; visualization; writing – original draft; writing – review and editing. **Colin R. MacKenzie:** Formal analysis; writing – review and editing. **Alexandros Dimitrakopoulos:** Data curation; supervision; writing – review and editing. **Francesca Pessanha‐Schlegel:** Supervision; writing – review and editing. **Daniel Kamp:** Supervision; writing – review and editing. **Leonhard Schilbach:** Conceptualization; supervision; validation; writing – review and editing.

## FUNDING INFORMATION

No funding or financial support was received for this case report.

## CONFLICT OF INTEREST STATEMENT

All authors declare no conflicts of interest related to the work presented.

## CONSENT

The patient gave written consent for anonymized publication of this case report.

## Data Availability

Data sharing is not applicable to this article as no datasets were generated or analyzed during the current study.
